# Social-emotional competence and early adolescents’ peer acceptance in school: Examining the role of afternoon cortisol

**DOI:** 10.1371/journal.pone.0192639

**Published:** 2018-02-20

**Authors:** Eva Oberle

**Affiliations:** School of Population and Public Health (The Human Early Learning Partnership), The University of British Columbia, Vancouver, BC, Canada; McMaster University, CANADA

## Abstract

The present study investigated the role of afternoon cortisol in social-emotional competence and peer acceptance in early adolescence. To date, research on basal cortisol activity and social development in childhood and adolescence has predominately focused on understanding maladjustment and dysfunction in development. The degree to which basal cortisol is also involved in positive adjustment and social functioning remains largely unexplored. A total of 154 early adolescents (46% female; *Mean age* = 11.26; *SD* = .65) from diverse ethnic backgrounds provided self-reports of perspective taking, peer reports of acceptance by classmates, peer reports of prosocial behaviors, and saliva samples to assess basal cortisol. As expected and in alignment with previous research, afternoon cortisol, perspective taking, prosocial behaviors, and peer acceptance were all positively correlated. Path analyses followed by bootstrapping analyses revealed that the direct path from higher afternoon cortisol to higher levels of prosocial behavior was fully mediated by perspective taking skills. The direct path from higher afternoon cortisol to peer acceptance was fully mediated by perspective taking skills and prosocial behavior. The findings are discussed within the broader context of previous research on cortisol and social adjustment in childhood and early adolescence. The practical relevance of the findings is considered.

## Introduction

Peer acceptance is an important developmental milestone in early adolescence; it has been linked to early adolescents’ wellbeing, resilience, and success in and outside of school [1–3]. Acceptance by peers in the school classroom is particularly important, given that early adolescents spend a substantial portion of their school day surrounded by and engaging with peers. Among the key personal characteristics that contribute to peer acceptance, social-emotional competencies (e.g., the ability to take others’ perspective, showing prosocial behavior) have been of particular interest for researchers and educators because they are malleable and can be fostered and taught in schools [4].

Overall, there is solid evidence for a positive link between social-emotional competencies and peer acceptance in early adolescence and other stages of childhood development [5–8]. The direction of the relationship, however, remains unclear; while some researchers have argued that social-emotional competencies drive acceptance by peers [9,10], others have taken the position that that being accepted by peers provides an opportunity for social interactions and thus enhances social-emotional competencies [11]. Furthermore, knowledge about the possibly contribution of third variables–such as underlying biological processes–remains limited. The present study addresses these limitations.

The goal of this study was to investigate the relation between social-emotional competencies (i.e., perspective taking, prosocial behavior) and peer acceptance in the classroom, and to explore to what extent afternoon cortisol activity may be involved in this relation as an underlying physiological process. Even though research on cortisol activity in relation to social-emotional competence and peer acceptance is scarce, a link is plausible. For instance, past research has shown that cortisol activity is connected to a range of social maladjustment indicators; specifically, lower cortisol levels have been linked to higher levels of aggression and antisocial behaviors in several research studies with children and early adolescents [12–15]. Both–aggression and antisocial behaviors–indicate a lack of social-emotional competencies, and are key predictors of peers rejection [16–19]. Given that cortisol activity is related to social-emotional dysfunction and peer rejection, it is possible that it also plays a role in *positive* social-emotional competencies and acceptance by peers. To date, only one preliminary study has explored the biological underpinnings of early adolescents’ positive social adjustment [13]; the authors found that afternoon cortisol specifically was positively related to peer acceptance and teacher-ratings of students’ prosocial behaviors. The present study aims to replicate and further expand on this finding by investigating possible pathways among basal afternoon cortisol, perspective taking, prosocial behavior, and peer acceptance in early adolescence.

There are several ways in which the present study advances research. First, a multiple-levels-of-analysis approach to child development [20] is employed by drawing from physiological markers, self-reports, and peer reports. Second, shifting the focus from basal cortisol linked to human dysfunction and maladaptation [12,14,21,22] to basal cortisol linked to social functioning and adjustment is consistent with perspectives of positive psychology and positive youth development [23,24]. Third, participants in the present study were typically developing early adolescents in their day-to-day school environment which presents a central ecological context for early adolescent development [25]; previous research in this field that has typically drawn from clinical participant samples, or was conducted in laboratory settings [15,26]. Last, the practical relevance of this research is apparent; it can inform the design of school-based interventions that target relationships with peers, enhance wellbeing, and prevent psychological and educational problems in school [9,27].

### Cortisol and social-emotional competence and functioning

Jointly investigating physiological processes and markers of social-emotional development and adjustment is critical for a more holistic understanding of child development [20]. The hypothalamic-pituitary-adrenal axis (HPA)–or stress axis–is a neurobiological system of strong interest for researchers; it maintains humans’ ability to respond to acute and prolonged changes in their environment, and involves the release of the stress hormone cortisol from the adrenal glands [28]. HPA-activity typically follows a diurnal pattern in which basal cortisol levels are high at awakening, increase further and peak within 20–45 minutes after waking, and then gradually decline across the day.

HPA-axis activity has been associated with the regulation of behaviors and emotions in several research studies [29]. A large number of studies has found lower basal cortisol concentrations in children and early adolescents who persistently display aggressive and antisocial behaviors [14,15,26,30–32]. A similar pattern was found in typically developing early adolescents with lower afternoon cortisol being associated with higher levels of reactive and proactive aggression [12]. However, in a few studies the opposite pattern was found with higher basal cortisol levels predicting externalizing behaviors [33,34]. Two explanations have been considered for why lower basal cortisol levels tend to be associated with higher levels of aggressive behaviors. The stimulation-seeking theory suggests that children with low basal cortisol levels seek stimulation to increase their arousal to a more optimal state [14,26,32]. Alternatively, the fearlessness theory suggests that low cortisol concentrations reflect fearlessness and a lack of inhibition, underlying aggressive behaviors [35].

The present study investigated to what degree basal cortisol in the afternoon plays a role in the link between social-emotional competence (i.e., perspective taking, prosocial behavior) and peer acceptance in the classroom in a sample of typically developing early adolescents. The focus was on basal cortisol in the afternoon because previous research has identified its positive relation to peer acceptance and teacher-rated prosocial behaviors [13], and its negative relation to peer reported reactive and proactive aggression [12,14,26]. Based on these studies, it was expected that afternoon cortisol would be positively related to perspective taking, prosocial behaviors, and peer acceptance. Consistent with previous research, positive interrelations were expected among perspective taking, prosocial behaviors, and peer acceptance [9,36–39]. A pathway model–from cortisol activity to peer acceptance, mediated through perspective taking and prosocial behavior–was developed and analyzed based on the following rationale: First, basal cortisol is one of the psychobiological processes that underlie behavior [20]; second, the ability to take others’ perspectives has been considered a foundation of prosocial behavior [40], and third, acting prosocially has been found to enhance peer acceptance [9]. An alternative model, in which the relation between prosocial behavior and peer acceptance was reversed (i.e., peer acceptance predicting prosocial behavior) [11] was also tested.

## Materials and methods

### Participants

Participants were 154 4^th^ to 7^th^ grade students (46% female) who were attending public elementary schools in an urban school district in Western Canada. All students had received written guardian consent and provided written assent for participation. Participation rate was 88%. Students were drawn from 6 classrooms located in 6 different schools. Schools were located in neighborhoods in which the median family income ranged from CA$ 64,895 to CA$ 96,648 (*Mean* = CA$ 75,310, *SD* = CA$ 11,699). The sample was representative of the ethnic diversity among families in the geographic region: 77% reported English as their language learned, 13% reported Cantonese or Mandarin, 5% reported Tagalog, and 3% reported Punjabi. The remaining students reported other languages. All participating students were proficient in reading and writing English. Seventy-three percent lived with both of their parents, 18% lived with either father or mother, and the remaining lived with other adults, such as relatives, grandparents, or in foster care.

### Measures

Participants completed a short demographic survey reporting their birth date, grade, ethnicity, sex, and family composition.

Acceptance by peers was assessed using a sociometric nomination procedure [3]. Students were provided with a roster of their classmates and asked to circle students in their classroom who they “would like to be in school activities [i.e., spend time] with” (a measure of peer acceptance). Students were instructed that they could circle as many or as few names as they liked. Only students who were participating in the study were included in the peer nomination measure. To control for classroom size (i.e., number of possible nominations), each participants’ peer acceptance score was derived computing the percentage of nominations received within the classroom by dividing the number of nominations received by the number of participating students in the classroom. Because data collection took place in the winter, students in each classroom were assumed to know each other well enough to make valid nominations. The same unlimited peer nominations procedure was used to assess early adolescents’ prosocial behaviors in the classroom [41]. Two types of prosocial behaviors were assessed in the present study: “shares and cooperates,” and “helps other kids when they have a problem”. The two prosocial behavior items were significantly correlated [*r*(151) = .86, *p* < .001], and were subsequently averaged to form a composite prosocial behaviors score.

Using the 7-item subscale of the Interpersonal Reactivity Index [42], children self-reported their ability to take someone else’s perspective on a 5-point Likert scale ranging from 1 (*not at all like me*) to 5 (*Always like me*). Example items are: “It’s easy for me to understand why other people do the things they do,” “Even when I am mad at someone, I try to understand how they feel.” Cronbach’s alpha indicated satisfactory inter-item reliability (α = .82). Reliability and validity of the IRI-subscales has been previously established in research with children and early adolescents [43,44].

**Fig 1:**
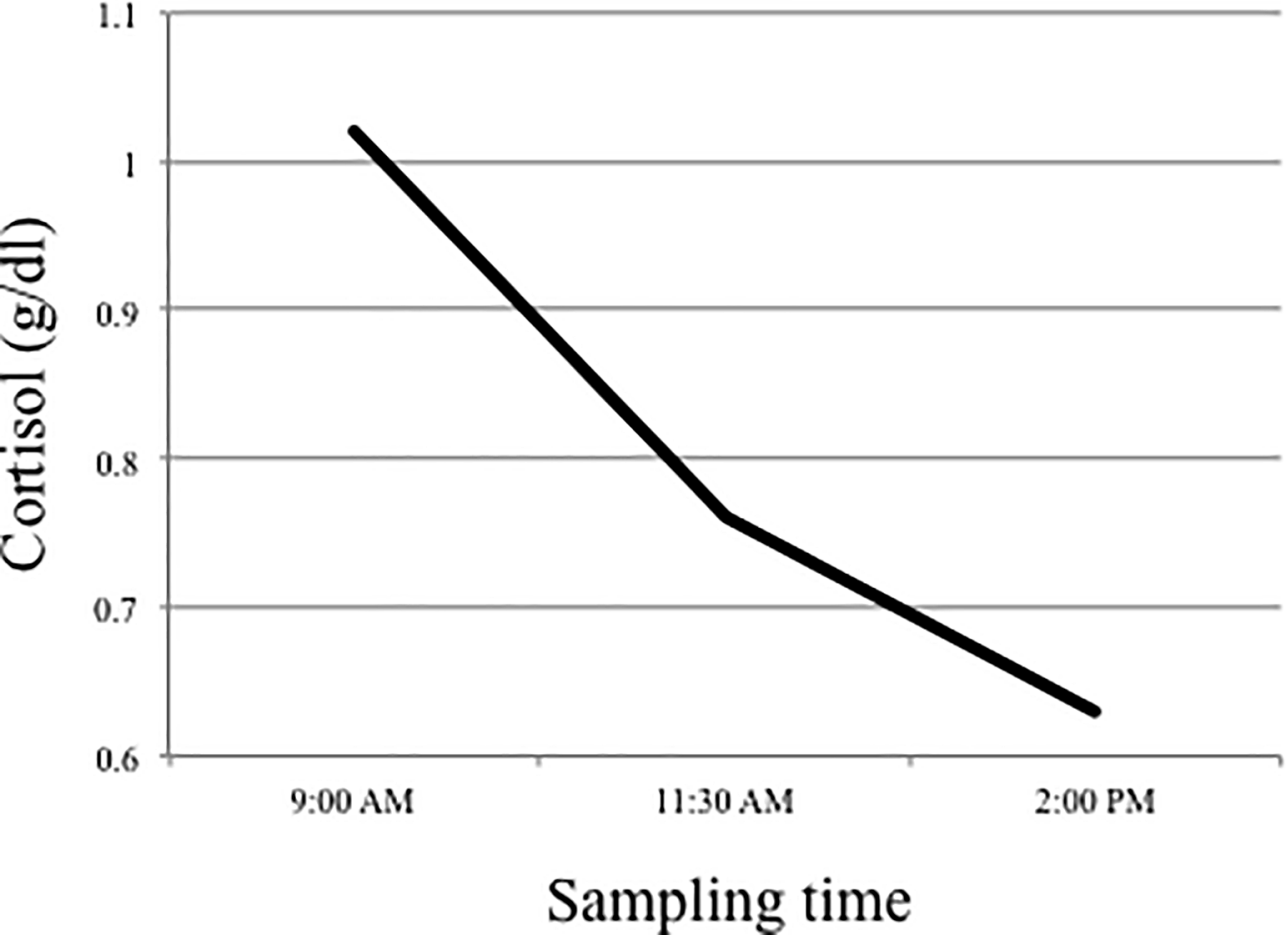
Basal cortisol output from morning to midday.

HPA-axis activity was assessed by measuring free cortisol in saliva at 9 a.m., 11:30 a.m., and 2:00 p.m. in the classroom setting on one day. Participants were instructed to avoid food and drink intake, and high physical activity prior to the saliva collection to avoid an artificial incline of cortisol. On the day of saliva collection, research assistants were present throughout school hours to guide the saliva collection, provide hands-on assistance, and ensure that students followed the outlined protocol. Samples were shipped to Clemens Kirschbaum's laboratory at the Dresden University of Technology in Germany for analyses of salivary cortisol. They were stored at -30° Celcius until all samples had been received. Upon completion of the study, samples were thawed, centrifuged and free cortisol concentrations in saliva were measured using a commercial chemiluminescence immunoassay (CLIA; IBL-Hamburg, Germany). Cortisol data were log-transformed for further analyses [45]. The cortisol measurement unit reported in the present study is g/dl. The cortisol means at each time point show a typical pattern of declining cortisol from early morning (*M* = 1.02, *SD* = .22) through mid morning (*M* = .66, *SD* = .22), to early afternoon (*M* = .63, *SD* = .21) in the present sample (Fig 1). Building on previous research [12,13], the focus of this study was basal cortisol in the afternoon.

Students' wake-up time on the day of data collection ranged from 6 am to 8:30 am (*Mean* = 7:20 am, *SD* = 37 minutes); it was not significantly related to children’s afternoon cortisol and was therefore not included in further analyses. Students’ self-reported their medication intake on the morning of cortisol collection. Several children reported various forms of vitamin supplements. One child reported cough medication. Two children reported ADHD medication. Given that their cortisol output was within the normal range around the sample mean, data were included in subsequent analyses.

### Procedure

Ethics approval was obtained from the University of British Columbia Behavioral Research Ethics Board (BREB), and the school district in which data were collected. Data were collected during two classroom visits by trained research assistants in the classroom setting in the half-way through the school year (i.e., January). All research assistants were graduate students who had participated in a faculty-led training workshop on data collection in classroom settings. Only students who had received parental consent and provided assent were included in the study. For the survey implementation, items were read out loud to control for different reading abilities among participants. Saliva samples were collected in the classroom via a cotton swab, which was then placed into a plastic tube labeled with the student’s study ID.

### Analytic procedure and preliminary analyses

Preliminary analyses were conducted and assumptions for path analysis were demonstrated. Descriptive and inter-correlations among all variables of interest are reported in Table 1. Missing data ranged from 0% to 8% across all variables. Listwise, 89% students had complete data on all variables; analyses were conducted on complete data (*N* = 137). As a rule of thumb, the recommend ratio of sample size to number of free parameters should be minimum 1:5 [46]; the present sample size to parameters ratio met this criterion. Path analyses were conducted using Mplus Version 7.4 [47]. Path analysis is an extension of the regression model and is used to test how a particular set of independent variables influence a particular dependent variable. Maximum Likelihood was used as estimator. The following fit indices and guidelines for model fit were used in accordance with established practices [48]: Root-mean-square error of approximation (RMSEA) values of less than .01, .05, and .08 were considered evidence for excellent, good, and mediocre fit. Comparative fit index (CFI) values greater than .95 were considered evidence for good fit. Standardized root-mean-square residual (SRMR) values less than .08 were considered evidence for good fit. The chi square test is also reported; a statistically non-significant chi square test indicates a good model fit [49].

**Table 1:** Intercorrelations, means, and standard deviations.

	Mean (SD)	1.	2.	3.	4.	5.
1. Age	11.26 (.65)	—				
2. Sex^1^	—	n/a	—			
3. 2 PM Cortisol	.63 (.21)	.161†	222**	—		
4. Perspective taking	3.21 (.75)	.093	.109	.233**	—	
5. Prosocial behavior	.49 (.20)	.025	.380***	.255**	.374***	—
6. Peer acceptance	.35 (.18)	.102	.103	.155†	.309***	.776***

† *p* < .10

*p < .05

**p < .01

***p < .001

^1^1 = boys

2 = girls

## Results

The bi-variate correlation analyses showed that as expected, afternoon cortisol, perspective taking, prosocial behavior, and peer acceptance were all positively interrelated (Table 1). Age was positively correlated with afternoon cortisol, and being female was related to higher cortisol and higher levels of prosocial behaviors. Based on these findings, age and sex were entered as control variables in subsequent analyses.

**Fig 2:**
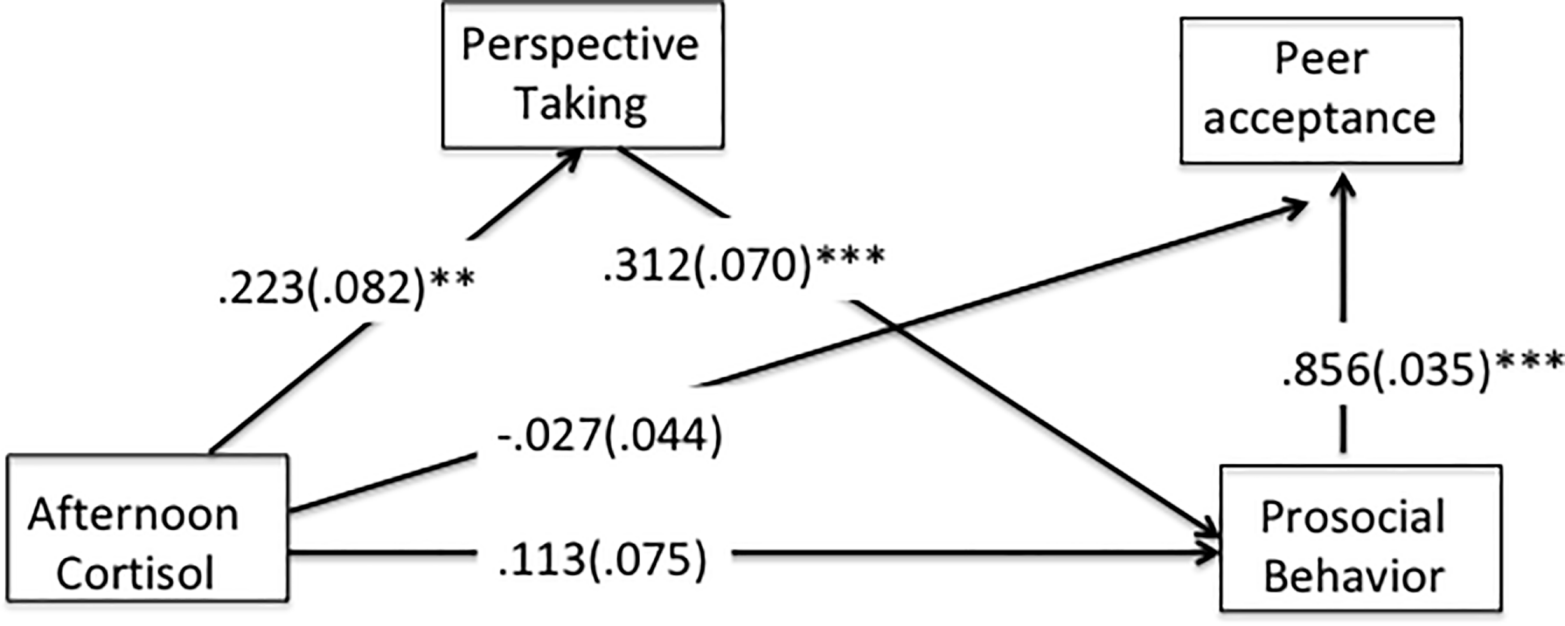
Direct and indirect pathways from afternoon cortisol to peer acceptance. Standardized coefficients are reported with standard errors in parentheses. ** < .01, *** < .001.

A path analysis was performed to test the hypothesized relationships (Fig 2). The path model had a good fit. It had a Chi Square of 0.529 (*df* = 3, *p* = .913), a CFI of 1.00, a SRMR of .01, and a RMSEA of 0.00 (RMSEA 90%-CI = 0.00 to 0.52). The model explained sixty-five percent of variability in peer acceptance (*R*^*2*^ = .650). All direct pathways along with standardized estimates and standard errors are reported. Two indirect pathways were tested and are reported: A) from cortisol to prosocial behavior through perspective taking, and B) from cortisol to peer acceptance through perspective taking and prosocial behavior. Significant pathways for control variables are reported in the text.

There were direct, positive, and significant pathways from afternoon cortisol to perspective taking (*standardized estimate* = .222, *p* = .007), from perspective taking to prosocial behaviors (*standardized estimate* = .312, *p* < .001), and from prosocial behaviors to peer acceptance in the classroom (*standardized estimate* = .862, *p* < .001). The direct pathways from afternoon cortisol to peer acceptance (*standardized estimate* = -.033, *p* = .538) and from afternoon cortisol to prosocial behavior (*standardized estimate* = .114, *p* = .125) were not statistically significant in the full model. Given the significant bivariate correlations between afternoon cortisol and peer rated prosocial behavior, afternoon cortisol and perspective taking, perspective taking and prosocial behavior, and the marginally significant correlation between cortisol and peer acceptance (see Table 1), and the subsequent drop to nonsignificance when jointly investigating the interrelations in the pathmodel, there was rationale for testing indirect effects of afternoon cortisol on prosocial behavior and peer acceptance.

A significant indirect effect was found from cortisol to prosocial behavior via perspective taking (*standardized estimate* = .069, *p* = .022). Bootstrapping analysis with 5000 samples was used to test for mediation [50,51]. The 95% CI ranged from .017 to .133, indicating that perspective taking fully mediated the pathway from cortisol to prosocial behavior. A further significant indirect effect was found from cortisol to peer acceptance via perspective taking and prosocial behavior (*standardized estimate* = .060, *p* = .023). In bootstrapping analyses, the 95% CI ranged from .014 to .113, supporting that the pathway from cortisol to peer acceptance was significantly mediated by perspective taking and prosocial behavior.

Regarding the control variables, girls had significantly higher acceptance levels than boys in the classroom (*standardized estimate* = .212, *p* < .001), were rated as more prosocial (*standardized estimate* = .316, *p* < .001), and had higher afternoon cortisol levels (*standardized estimate* = .238, *p* = .002). The direct path from age to afternoon cortisol was significant and positive (*standardized estimate* = .182, *p* = .020), and the path from age to peer acceptance was marginally significant (*standardized estimate* = .081, *p* = .098).

An alternative model in which the positions of peer acceptance and prosocial behavior were reversed in the model while cortisol activity and perspective taking remained in their positions (i.e., from cortisol to perspective taking, to peer acceptance, to prosocial behavior), was tested. Results indicated a poorer model fit compared to the previous model with a marginally significant Chi Square of 6.808 (*df* = 3, *p* = .078), CFI of .98, SRMR of .03, and a RMSEA of 0.09 (RMSEA 90%-CI = 0.00 to 0.18); these fit indicators supported rejecting the alternative model.

## Discussion

The goal of this study was to investigate the relation between social-emotional competencies and peer acceptance in the classroom, considering the underlying role of afternoon basal cortisol. The role of cortisol in positive social functioning in day-to-day settings (e.g., school) remains largely unknown; previous research has taken an almost exclusive focus on the cortisol activity in relation to social dysfunction and maladaptation in child development [15,26,52]. A shift towards physiological processes that are linked to positive development in early adolescence is much needed [53].

There were two key findings in the present study. First, as expected, afternoon cortisol, perspective taking, prosocial behavior, and peer acceptance were all positively interrelated. This finding is consistent with previous research that has established positive relations between social-emotional competencies and peer acceptance in childhood and early adolescence [5,8,39,54]. It is in accordance with the positive relation between afternoon cortisol and social functioning found in a previous study [13]. Finding that higher afternoon cortisol was linked to better social-emotional functioning is further consistent with research that has revealed the opposite pattern when examining the relation between cortisol social dysfunctioning (i.e., lower basal cortisol predicted higher levels of dysfunctioning previous research) [12,14,15]. A possible explanation comes from the theory that blunted basal cortisol indicates understimulation, and drives aggressive behaviors in children and adolescents in order to reach comfortable arousal level [26,32]. Higher cortisol levels within a normative rage may hence indicate optimal arousal and thus facilitate social-emotional functioning.

A second key finding in the present study was that the link between afternoon cortisol and prosocial behavior was fully mediated by perspective taking, and the link between afternoon cortisol and peer acceptance was fully mediated by perspective taking and prosocial behavior. This suggests that higher basal afternoon cortisol in the present study contributed to better perspective taking, which in turn contributed to increases in prosocial behaviors and higher levels of peer acceptance. This is an interesting and novel finding that can be understood by considering the core skills that are involved in successful perspective taking. In fact, taking someone else’s perspective requires competence in social understanding, cognitive functioning and self-regulation [55–59]. For example, it entails understanding what another person is feeling or experiencing, distinguishing their experience from one’s own experience, and switching back and forth flexibly between the two perspectives [60]. Switching between perspectives in particular taxes self-regulatory abilities, such as inhibitory control and cognitive flexibility [61]. Self regulatory abilities, such as emotion regulation, in turn have been linked to basal cortisol in children and adolescence in previous research [12,29]. Self-regulatory skills have also been linked to the effective downward regulation of cortisol elevation in response to stress exposure in laboratory experiments with early adolescents [62].

In addition to the contribution to research, identifying perspective-taking as a key competency that connects physiological and social-emotional functioning is also of practical relevance. Several school-based social-emotional learning interventions have shown that perspective-taking can be promoted and enhanced in childhood and early adolescence, and that doing so contributes to positive social, behavioral, and academic outcomes [63–66].

Concluding, the present study advances previous research by suggesting that physiological processes and positive social-emotional functioning are interconnected in early adolescents’ development in their natural day-to-day environment. It extends previous research that has consistently found a link between cortisol activity and social-emotional maladjustment. The mediating role of perspective taking that accounted for the link between cortisol and prosocial behavior, and the link between cortisol and peer acceptance, was emphasized in the present study.

### Limitations

Despite the novel findings that emerged of this research, several limitations need to be considered. The main limitation was that due to the financial cost associated with sampling and analyzing saliva from a larger number of early adolescents in schools, cortisol samples were collected on one day only. Even though the researchers put into place clear classroom guidelines for the day of data collection to limit biases in the data (e.g., teachers were asked to refrain their students from eating drinking, and exercising before saliva sampling, and no physical education classes took place on the sampling day) it cannot be ruled out that particular classroom-specific events that were unknown to the researchers, took place (e.g., high levels of conflict with peers or the teacher). A further limitation was that there may be other confounding variables that were not measured in the present study (e.g., early adolescents’ socioeconomic background). Because the present study is the first of its kind, findings need to be considered preliminary. Last, the present study was cross sectional and causal interpretations cannot be made based on the study design.

### Future directions

Sampling cortisol on several consecutive days and averaging the daily mean values is critical because it controls for day-to-day and situational variability in cortisol levels. Future research also needs to take into account pubertal status and socioeconomic family background as control variables. Research needs to investigate to what degree the patterns identified in the present study may differ across developmental stages, and at different time points throughout the school year, for instance when young people are still building friendships in the beginning of the year. Future research needs to investigate the alternative path model, in which peer acceptance is a precursor of prosocial behavior. While the alternative model did not show a good fit in the present study, it is possible that increases in prosocial behavior due to increases in peer group belonging would only be detectable in longitudinal research.

## Supporting information

S1 File Supporting.data.Cort.Prosocial. (SAV)
